# Management of low back pain in general practice – is it of acceptable quality: an observational study among 25 general practices in South Tyrol (Italy)

**DOI:** 10.1186/1471-2296-14-148

**Published:** 2013-10-04

**Authors:** Giuliano Piccoliori, Adolf Engl, Doris Gatterer, Emiliano Sessa, Jürgen in der Schmitten, Heinz-Harald Abholz

**Affiliations:** 1 Accademia Altoatesina di Medicina Generale (AcAMG), Bolzano, Italy; 2 Statistics, Agenzia Regionale di Sanità Toscana, Florence, Italy; 3 Institute of General Practice, Medical Faculty, Heinrich-Heine-University, Düsseldorf, Germany

**Keywords:** Low back pain, General practice, Guideline adherence, Diagnostic imaging

## Abstract

**Background:**

There are several guidelines dealing with the management of low back pain (LBP), but only few studies on the quality of care provided within General Practices as judged against those guidelines.

The objective of this study is to analyse the management of LBP in Italian General Practice and compare it with guideline recommendations.

**Methods:**

In this observational study, all patients visiting their General Practitioners (GPs) for treatment of LBP within a 8-week period were monitored for at least four weeks with regard to symptoms and diagnostic and therapeutic interventions. Management of LBP was judged by pre-defined quality indicators based on guideline recommendations.

**Results:**

Twenty-five of 114 eligible GPs participated in the study, representing a total of 43,012 registered patients. Of the 475 patients complaining of LBP and monitored for four weeks, 55.8% were diagnosed as having acute lumbar pain, 13.5% chronic lumbar pain, 17.1% acute sciatica, and 12.6% chronic sciatica; 76.0% underwent no technical investigations, 21.7% underwent x-rays, 5.5% MRI and 4% CT scans; 20.4% were referred to secondary care; 93.3% of all patients received some medication. In those receiving a medication, in 88.3% it was an NSAID, in 6.3% Paracetamol, in 10.4% Paracetamol combined with Codeine, and in 9% a muscle relaxants. When physiotherapy was prescribed (17,1%), it was mostly massage. Hardly more than 50% of GPs (partially) followed locally established guidelines, while the remainder seemed not to follow guidelines at all.

**Conclusions:**

Our study reveals gross deviations of GP management of LBP from current guidelines and points to two different types of deviators: those who partially follow guidelines, and those who do not follow them at all. Further research should evaluate whether these two types of deviation are best addressed by different foci of education, i.e. on knowledge versus attitudes, respectively.

## Background

Low back pain (LBP) is a common ailment among patients visiting GPs [1]. The annual prevalence of LBP is given as 15–45% [2,3], the annual incidence as 5% [3-5]. Among patients suffering from acute LBP, 5–10% will develop chronic LBP [6-14]. Studies of the last two decades have shown that traditional treatment of LBP, focusing on injections and (bed)rest, may contribute to chronification and its devastating individual and macro-economic sequelae. Current guidelines [15], including those in use in South Tyrol [16,17], suggest to recommend paracetamol as the analgetic drug of first choice, followed by non-steroidal anti-inflammatory drugs (NSAIDs). Because of the mostly benign nature of the condition, only in few cases diagnostic imaging or specialist involvement are indicated.

However, there is still a lack of available data from primary care sources on the epidemiology of care for LBP [18-24].

The aim of this paper is to show how LBP is managed within Family Practices in a gate-keeper system in South Tyrol in Northern Italy, focusing on diagnostic investigations and treatment, and comparing these procedures to actual guideline recommendations.

## Methods

### Design and recruitment of GPs / patients

For this observational prospective cohort study, all 114 General Practitioners (GPs) based in Bozen (the capital of South Tyrol) and the surrounding rural area were invited by post to participate. They were informed that this would include the monitoring of all patients coming in for LBP treatment during a defined period of time (i.e., 8 weeks), and that they would receive 30 euros per patient. This “pay for inclusion” system was designed to ensure that the GPs taking part would include *every* LBP patient consecutively visiting the practice. Additionally, GPs were phoned three times in this period of eight weeks to support a complete inclusion of all eligible patients.

To be recruited to the study, GPs had to be working for at least four years in a practice so as to confine the study to the more experienced GPs, and thus arrive at a conservative estimate of guideline adherence. Financial constraints limited our sample to the first 25 GPs who applied to take part and fulfilled the inclusion criteria.

All patients were monitored and documented by GPs for at least four weeks following their inclusion in the study. We report here data obtained in the first 4 weeks of monitoring of the included patients. For a subset of patients that we were able to monitor for at least 9 weeks (n = 198), we report the number of further visits with their GP.

A more detailed account of the study methods and its epidemiological results has been reported elsewhere [18].

### Definitions

The participating GPs were provided with the following definitions:

**Low back pain**: Back pain from the low costal arch to the gluteal muscles which is not caused by any secondary condition (herpes zoster, renal colic, etc.).

**Acute low back pain**: Pain lasting less than 12 weeks, or recurring LBP following a pain-free interval of at least 6 months.

**Chronic (recurring) low back pain**: Pain lasting 12 weeks or longer or recurrences within less than 6 months.

**Lumbar pain**: No neurological (senso-motor) deficits; Pain does not radiate below knee; pain not restricted to a specific dermatome.

**Sciatica**: Dermatome-related pain to area below the knee; often more intense than in the lumbar region; pins and needles or numbness; occasional muscular weakness; reduced reflexes.

**Complex LBP (“red flags”)**: senso-motor deficits; pain due to tumour, metastasis, fracture or infection.

### Inclusion and exclusion criteria

Patients aged at least 18 years were to be *included* if they visited the practice with either

a) acute LBP or

b) chronic (recurring) LBP.

Patients who had received treatment for their current condition prior to the study were *excluded*. Patients whose LBP was caused by a malignancy or fracture (so-called “red flags”) were *also excluded* whenever this was detected, partly to arrive at a relatively homogenous sample and partly because this subgroup was expected to be very small, not allowing a subgroup analysis for these patients.

### Documentation

GPs were issued a specifically designed questionnaire (spreadsheet in excel format) in which they documented each patient’s sociodemographic baseline data, type of LBP, pain level on a scale of 1 to 10 (score elicited from the patient), diagnostic procedures, therapeutic interventions and any referrals to secondary care. The questionnaires were to be filled out, at baseline and at each further visit. In order to keep workload low and participation / compliance high, GPs were only asked to document the patients they included. Naturally, patients with unobvious “red flags” were initially documented but excluded in the later course whenever the “red flags” became apparent.

### Compliance with guidelines

In order to define the extent to which GPs complied with guidelines, we developed an assessment instrument based on the main diagnostic and therapeutic recommendations (quality indicators) of the two guidelines in local use [16,17]. We also had to establish the percentage of patients with LBP visiting a practice who should receive the care proposed by the quality indicator (*proportion of quality indicators reached*). This assessment instrument follows the principles of “pay-for-performance” systems in use in the UK and discussed for example by the American College of Physicians [25].

Our assessment instrument was based on the following five quality indicators and the required percentage of patients who should receive the care proposed:

1 For *acute lumbar pain* no more than 10% of patients should undergo an X-ray during their first visit.

2 For *acute sciatica* no more than 10% of patients should undergo an X-ray, CT and/or MRT during their first visit.

3 For *lumbar pain or sciatica* no more than 10% of patients should be referred to an orthopaedic specialist or neurosurgeon during their first visit.

4 For *lumbar pain or sciatica* no more than 5% of patients should be given an intramuscular injection of a NSAID.

5 For *lumbar pain or sciatica* not less than 30% of patients should be prescribed paracetamol alone or in combination.

### Statistics

Data are presented as percentages, while groups were compared using chi-square Pearson test. Statistical analyses were performed with STATA for Windows 10.0 (StataCorp.LP, College Station, TX, USA).

### Ethical approval

In Italy it is not necessary to secure ethical approval for the analysis of pseudonymized administrative data.

## Results

Of the invited 114 eligible GPs in this region, the first 25 GPs applying for participation were chosen. They served a total of 43,012 registered patients. On average, there were 1,720 registered patients per GP (minimum 718, maximum 2395). Responder analysis of the 25 participating GPs compared with the residual 89 GPs showed no difference with regard to gender, age, years of experience, or number of registered patients (data not shown).

During the initial eight-week period, the 25 GPs recorded the visits of 487 patients complaining of LBP as defined by the inclusion criteria. In the course of the diagnostic workup, 2 patients with malignancies and 10 suffering from osteoporotic fractures had to be excluded from the sample (see exclusion criteria). The remaining sample (n) of 475 patients was monitored for the subsequent four weeks. Each GP enrolled on average between one and five patients per week.

### Clinical presentation

The average age of these patients with LBP was 55,3 (SD 17,8; min 18, max. 93) years, and 57% of them were female. Overall, 72.5% of patients with LBP saw their GP only once within the initial four-week period, 22.3% visited twice, and 5.2% three times or more. In a subgroup of patients whom we were able to monitor for at least nine weeks (n = 198), only 16% visited their GPs again in the additional five weeks adjoining the 4 weeks monitoring period for all patients.

Of the 475 patients 265 (55,8%) were diagnosed with acute lumbar pain -and 64 (13,5%) as chronic lumbar pain; 81 (17.1%) were diagnosed as acute and 60 (12.6%) as chronic sciatica, 5 (1%) were missings.

Pain was assessed by the patient using an NRS (numerical rating scale, rating the pain between 0 = no pain and 10 = maximal pain). At the time of the first visit the average level of pain was 6.0 (SD 0,7; max. 7,7, min. 4.5). Patients who visited their GP only once, i.e. at the first contact, rated themselves on average at a non-significant slightly lower pain level (5.6 on the NRS) than the rest of those who visited the GP several times. Of the 131 patients who saw their GP more than once during the four-week follow-up, the average level of pain fell to 3.2 (SD 1.8; max 6.1., min. 0,0) over the period between first and second visit.

### Diagnostic interventions and referrals to secondary care

Seventy-six percent (n = 361) of all patients received no technical (including laboratory) investigations during the four-week follow-up period. 21.7% underwent an x-ray, 5.5% an MRI scan and 4% a CT scan. Few patients received many tests; in those getting at least one test, the average number of tests was 1,2 (SD 1,3; min.1, max.8).

20.4% of the patients were referred to secondary care: 11.6% to an orthopaedic surgeon; 6.5% to a neurosurgeon, and 2.3% to a specialist doctor for physical therapy (a medical specialisation in Italy).

There were a number of differences between treatments of the two differential types of low back pain (lumbar vs. sciatic) with regard to diagnostic interventions and referrals (Table 1). Significantly more patients with *acute* sciatica (49.4%) were referred to secondary care and/or received diagnostic interventions than those with acute lumbar pain (24.2%, p < 0.05). The same trend held for *chroni*c LBP when sciatica and lumbar pain were compared (data not shown).

**Table 1:** Requested investigations and referrals to secondary care for different acute LBP types within four weeks from initial visit (n = 346)

**Investigation**	**Acute lumbar pain (n = 265)**	**Acute sciatica (n = 81)**	**p-value**
X-ray	19.2%	21.0%	0.72
MRI scan	1.5%	13.6%	<0.05
CT scan	0.4%	13.6%	<0.05
Blood test	3.0%	1.2%	0.35
**Referrals to**			
Orthopaedic surgeons	6.8%	13.6%	0.06
Neurosurgeons	0.0%	23.5%	<0.05
Specialists for physical medicine	1.5%	2.5%	0.60
At least one investigation requested	24.2%	49.4%	<0.05

### Variations among GPs

A striking discrepancy among individual GPs was found in terms of referring patients *with acute lumbar pain*. Overall, 14% (37/265) of patients with lumbar pain were referred for an x-ray during their first consultation, but six GPs referred more than 20% (17/52) of their patients during the first consultation, including one who requested x-rays for 57% (3/5) of his patients. Five GPs, on the other hand, did not request an x-ray at all (0/30), and another five requested x-rays for fewer than 10% (5/84) of their patients (p = 0.004 for variability).

The average levels of pain recorded by patients across all practices were statistically similar among these groups (data not shown).

Compared to patients with acute lumbar pain (of whom only one received a CT scan and only three received MRI scans following the first consultation; together 2.1%), patients with acute *sciatica* received a CT scan in 7.9% (6/81) and an MRI scan in 6.1% (5/81) of cases after the first consultation.

The discrepancies among GPs concerning the care of sciatica patients seemed remarkable: 15 did not refer any patients for a CT scan (0/38), while five did so in more than 30% (4/11) of cases during the first consultation; however, these differences did not reach statistical significance (p = 0.290). The average levels of pain among these two groups were statistically similar (data not shown), so differences in pain levels do not explain these discrepancies. Twenty GPs did not request an MRI scan for any of their patients (0/65), but five sent almost a third (31,8%, 5/16) of their patients with sciatic pain for an MRI scan (p = 0.063).

### Treatment

#### *Physiotherapy*

Only 17.1% of all patients received any form of physical therapy. Of these, massageconstituted two thirds of all prescriptions for physiotherapy, significantly more than prescriptions for regulated exercise.

### Medication

Only 6.7% of all patients were prescribed no medications at all within the four-week monitoring period. Of those patients who were prescribed at least one medication, 88.3% received an NSAID, 6.3% paracetamol, 10.4% paracetamol in combination with codeine, 9% muscle relaxants, 5.2% steroids and 2.9% tramadol (Table 2).

**Table 2:** Prescribed medications within the first 4 weeks (n = 475)

**Medication**	**n**	**%**
One or more medications	443	93.3
**Of these (100%)**		
NSAID	391	88.3
Paracetamol + codeine	46	10.4
Muscle relaxant	40	9.0
Paracetamol	28	6.3
Steroids	23	5.2
Tramadol	13	2.9
Gabapentin	10	2.3
Benzodiazepin	9	2.0

More than one answer allowed.

More than one medication per patient possible.

NSAID: non-steroidal anti-inflammatory drugs.

We found that the prescription rate of some medications within each category of LBP varied significantly. More patients suffering from *acute sciatica* were prescribed steroids (p < 0.05) and tramadol (p < 0.05) than patients with *acute lumbar pain*. The prescription rate of NSAIDs did not vary significantly (p = 0.71). 17% of patients with acute sciatica were prescribed muscle relaxants (Table 3).

**Table 3:** **Pharmacological treatment in patients with *acute *LBP (n = 346)**

**Medication**	**Acute lumbar pain (n = 265)**	**Acute sciatica (n = 81)**	**p-value**
No medication	4.9%	6.2%	0.65
Paracetamol	5.7%	9.9%	0.18
Paracetamol + codeine	8.7%	8.6%	1
NSAID	85.3%	]90.1%	0.26
Tramadol	1.1%	7.4%	<0.05
Muscle relaxant	6.8%	17.3%	<0.05
Benzodiazepine	2.6%	1.2%	0.46
Steroids	3.4%	9.9%	<0.05
Gabapentin	0.4%	3.7%	<0.05

Data relate to all consultations, not just the first consultation.

More than one medication per patient possible.

NSAID: non-steroidal anti-inflammatory drugs.

Eighty-five percent of patients with acute lumbar pain received an NSAID preparation during their course of treatment. Most were prescribed an NSAID during their first consultation. Some patients were also prescribed tramadol and/or paracetamol along with an NSAID during their first visit or received them at a later consultation.

Two GPs prescribed paracetamol (alone or in combination) in more than 50% of all cases while another eleven GPs prescribed it in less than 10% of their patients (p =0.000).

### Route of administration (NSAID)

In 25.6% of all patients receiving NSAIDs at the first consultation these were administered as an intramuscular injection. A number of these patients received repeated injections. There were differences in the route of administration within each category of LBP. Patients with chronic LBP were much less likely to receive an intramuscular injection than those with acute symptoms (p < 0.05). Four GPs provided no intramuscular injections at all, while five others used this as the preferred application (more than 50% of primarily given NSAID) (Table 4). Many GPs combined both methods of application.

**Table 4:** **Modes of NSAID application at first consultation in patients with *acute *LBP (n = 346)**

**Mode of application**	**Acute lumbar pain (n = 265)**	**Acute sciatica (n = 81)**	**p-value**
**NSAID**			
Oral	69.3%	60.6%	0.11
Intramuscular	17.0%	30.9%	<0.05
Intramuscular and oral	8.7%	4.3%	0.15
Intravenous	2.7%	3.2%	0.79
Transdermal	2.3%	1.1%	0.45

More than one medication per patient possible (number of prescribed medications: n_p_=394).

NSAID: non-steroidal anti-inflammatory drugs (note: in Italy also available for intravenous and transdermal application).

### Quality of care

In applying our pre-defined assessment instrument in order to summarise the quality of care (see Methods), it was found that among the 25 participating GPs, three failed in all five of the quality indicators, and eight failed in four of them. We denominate these 11 GPs as *non-compliant with the guidelines.*

The two GPs fulfilling all five quality indicators were denominated as *fully compliant*.

Another four GPs fulfilled three of the five quality indicators, and eight fulfilled just two. These were denominated as *intermediate* in terms of their *compliance with the guidelines* (N = 12). The groups of *fully* or *intermediately compliant* GPs compared to those termed *non-compliant* did not differ significantly with regard to the following characteristics: age and gender of both GPs and patients; patients’ pain-scale scores, and number of patients cared for by a GP (data not shown).

## Discussion

This study among a sample of 475 patients consecutively consulting their South Tyrolian GP with LBP demonstrated that hardly more than half of the participating GPs were likely to follow local guidelines on LBP whereas the other half seemed not to follow guidelines at all. For example, 21.7% of all patients underwent x-rays, 20.4% were referred to secondary care, and of the 93% who were prescribed medication, 88% received NSAID whereas the first-line recommendation paracetamol was given in only 10%.

### Prevalence and severity of LBP, repeated consultations

Our study confirms that in the studied group of GPs and their patients, repeated visits in the following month after consultation for low back pain are the exception rather than the rule. Other studies within General Practice have found higher rates of second or third consultations [6,8,26]. But – judging by the small samples and relatively long corresponding enrolment periods in those studies – they may have been selective in terms of inclusion. We believe that our figures reflect the observed setting accurately, i.e. that we have not lost patients consulting “other doctors”, because in Italy registered patients are only insured to consult primarily their GP with whom they are registered, whereas direct contact to specialists is not covered (except for out-of-hours visits or accident and emergency services).

The finding of low re-visiting rates suggests “minor” LBP episodes. But the opposite seems to be the case: The percentage of sciatica cases within the total number of patients with LBP is remarkably high (nearly 30%). Other studies have reported a much lower occurrence of sciatica, sometimes only 5% [2]. But there are also studies that identify rates comparable to our own findings [27]. A factor supporting the validity of our data is that the GPs in our study – in contrast to all other studies in the ambulatory setting – received a list of definitions relating to the various types of LBP beforehand.

The constellation of low re-visiting rates on the one hand, and the high rate of sciatica (i.e. complicated LBP) on the other, is relevant to the interpretation of our results with regard to diagnostic and treatment measures. Considering the two relevant guidelines on LBP in local use, these findings make over-treatment more likely than under-treatment.

### Guideline adherence

In line with current guidelines, 76.0% of the study patients were not referred for technical diagnostic investigations. However, contrary to these guidelines, 14.3% of patients with *acute* lumbar pain and 16.7% with *acute* sciatica were referred for an x-ray during their first consultation. In addition, referrals for CT or MRI scans were requested in 7.9% and 6.1% respectively of the acute sciatica cases during the first consultation, which is also higher than expected, judging by the guidelines based on the epidemiology of symptomatic acutely-slipped discs truly warranting radiological imaging.

Only a few GPs prescribed paracetamol, even though all guidelines in local use [16,17] recommend this as the first option. Most people in Italy see paracetamol as a means to combat fever and are unlikely to use it as a remedy for LBP. If combined with codeine, paracetamol requires a prescription.

Furthermore, three quarters of all patients with low back pain received an NSAID. Most of those with high pain levels received intramuscular injections, which is not recommended in guidelines. One possible explanation for this adherence to obsolete treatment procedures is – referring to the results of a previous study in Germany [28] – that GPs are still over-reliant on the presumed placebo effect of injections.

We identified five other studies on the treatment of LBP in general practice, employing a retrospective or prospective approach. One from Germany [26] had a highly selective population, two from the Netherlands showed no obvious selection of patients [20,21], one very small study from Ireland was based on just 59 patients and eight GPs [22], and a large study was carried out in Australia [23]. However, comparison with these studies is limited owing to the widely varying characteristics of the patient samples and discrepancies in the lengths of observation periods.

Approximately half of the participating GPs in our study treated their patients more or less according to the guidelines, while the remainder provided care that was hardly or not at all compliant with local guidelines. In particular, some GPs relied too much on diagnostic imaging (X-ray, CT, MRI) during the first consultation – even for those patients who only suffered from lumbar pain. We also find this trend in the other studies cited above, but mostly to a lesser extent. Possible explanations include the wide time spans between the above cited studies and the different medical cultures within the countries where the studies took place.

The other main finding of our study was that paracetamol was not prescribed often enough either alone or in combination with codeine, a phenomenon also found in the other five studies.

Our study was unique in identifying the relatively high percentage of intramuscular NSAID injections prescribed. The other German study [19] did not investigate this. However, along with this other German study [19] and the Irish study [22], we found that physiotherapy was carried out in a low percentage of cases – and it was usually only massage, not regulated exercise which was prescribed.

Our finding that guidelines were not being followed could represent a more general scepticism on the side of the GPs towards guidelines, particularly in the area of LBP where more individualized care may be assumed to be necessary [21,29]. However, in a study on the care of patients with chronic heart failure in South Tyrol, we found the opposite trend: there, most GPs followed guideline recommendations quite rigidly [30].

This observation suggests that the cultural concept or notion of LBP should be considered when compared to heart failure, for example. In most cases LBP cannot be regarded as the result of concrete physical injury – except “stress and strain”. Some authors regard LBP as a psychosomatic and culturally defined condition, or at the very least as an illness strongly influenced by psychosocial factors [31]. Additionally, it is a symptomatic illness that puts pressure on the doctor to do something. Fully aware that medication and exercise are not likely to effect “healing” quickly, the GPs may feel compelled to take short-term measures, which could take the form of diagnostic interventions.

If this were true, a merely rational approach– as stipulated in the guidelines – is not sufficient to change the doctors’ behaviour. Rather, an approach that focuses more on the emotional aspects of the relationship between patient and doctor would seem more promising. Thus, we suggest evaluating educational interventions aimed at encouraging doctors to address perceived pressures and anxieties openly and to inform patients about the benign nature of the condition. In an interventional study aimed at changing the behaviour of GPs prescribing antibiotics for acute cough, such an approach was shown to be effective [32].

### Strengths and limitations

One of the study’s strengths comes from our efforts to include *all* patients seen by their GPs in an attempt to reduce any selection bias. Another strength is the decision to provide criteria to GPs beforehand that defined the different kinds of LBP. Moreover, thanks to an intense data monitoring, rigorous phone tracking in case of missing data, and an extraordinary commitment of the participating physicians who know each other and cooperate well in this small community of GPs in South Tyrol, we achieved a high data quality (few missing data).

One obvious limitation is that, due to financial constraints, our system of enrolling GPs – taking on only the first 25 who applied and fulfilled the inclusion criteria – could have favoured those who were most interested in the subject matter of our study and thus created a bias. Despite this, our results hardly draw a picture of perfect GPs happily following guidelines, and this probably suggests that in reality the average GP deviates even further from guideline recommendations than our findings suggest.

Also, we cannot know for sure that the GPs included all consecutive patients with LBP. However, we did not attempt to control the inclusion process since we felt that this would be too much interference, if at all feasible, and eventually counter-productive.

Further, our assessment instrument described in the Methods section was not a validated instrument since we could not identify one at the time. However, we found it to be a pragmatic and transparent approach for providing insights into what we consider the “right” or “wrong” courses of action, and it gives an approximate assessment of whether guidelines are followed. Its main shortcoming is that it fails to address questions relating to whether each individual patient is receiving the optimal treatment. But that would be difficult to judge without all the patients being seen again by a qualified “controlling physician” who could bring together the individual clinical situation with the corresponding recommendations of the study physician.

Another limitation of our study is that we were only able to assess compliance to guidelines using plausible assumptions (the proportion of cases achieving certain quality indicators) and were therefore unable to analyse on the basis of individual “cases”.

## Conclusions

Our study reveals gross deviations of GP management of LBP from current guidelines, resulting in likely over-diagnosis with imaging techniques, over-treatment with NSAIDs, and over-utilisation of an obsolete mode of NSAID application, i.e. intramuscular injection. All three guideline deviations are potentially harmful.

The data point to two different types of deviators: those who partially follow guidelines, and those who do not follow them at all. Further research should evaluate whether these two types of deviation are best addressed by different foci of education, i.e. on knowledge versus attitudes, respectively.

## Abbreviations

CT: Computer tomography; GP: General practitioner; LBP: Low back pain; MRI: Magnetic resonance imaging; NSAID: Non-steroidal anti-inflammatory drugs.

## Competing interests

The authors declare that they have no competing interests.

## Authors’ contributions

GP participated in the design, was the leading researcher in South Tyrol realizing the study and its data-analysis, and worked on the publication. AE arranged for the funding, participated in the conception and worked on the publication. DG worked together with GP on the realization of the study in South Tyrol. ES gave statistical advice and analysed data statistically. JidS participated in the conception of the study, contributed to the draft and finalised the manuscript. HHA conceived of the study, participated in its design, supervised both execution and analysis, and helped to draft the manuscript. All authors read and approved the final manuscript.

## Pre-publication history

The pre-publication history for this paper can be accessed here:

http://www.biomedcentral.com/1471-2296/14/148/prepub

## References

[B1] Hart LG Deyo RA Cherkin DC Physician office visits for low back pain. Frequency. Clinical evaluation, and treatment patterns from a U.S. National survey Spine 1995 20 11 19 10.1097/00007632-199501000-00003 7709270

[B2] Salaffi F De Angelis R Grassi W Revalence of musculoskeletal conditions in an Italian population sample: results of a regional community-based study. MArche pain prevalence; INvestigation group (MAPPING) study Clin Exp Rheumatol 2005 23 819 828 16396700

[B3] Andersson GBJ Frymoyer JW The epidemiology of spinal disorders The Adult Spine: Principles and Practice 1997 2 New York: Raven Press 93 141

[B4] Raspe H Kohlmann T Kreuzschmerzen - eine Epidemie unserer Tage? (Low back pain – an epidemic to-day?) Dtsch Ärztebl 1993 90 2920 292524089288

[B5] Deyo RA Weinstein JN Low back pain N Engl J Med 2001 344 363 370 10.1056/NEJM200102013440508 11172169

[B6] Van den Hoogen HJ Koes BW Eijk JT V Bouter LM Deville W On the course of low back pain in general practice: a one-year follow-up study Ann Rheum Dis 1998 57 13 19 10.1136/ard.57.1.13 9536816 PMC1752458

[B7] Schiottz-Christensen B Nielsen GL Hansen VK Schodt T Sorensen HT Olesen F Long-term prognosis of acute low back pain in patients seen in general practice: a one year prospective study Fam Pract 1999 16 223 232 10.1093/fampra/16.3.223 10439974

[B8] Croft PR Macfalane GJ Papageorgiou AC Thomas E Silman AJ Outcome of low back pain in general practice: a prospective study BMJ 1998 316 1356 1359 10.1136/bmj.316.7141.1356 9563990 PMC28536

[B9] Henschke N Maher CG Refshauge KM Herbert RD Cumming RG Bleasel J Prognosis in patients with recent onset low back pain in Australian primary care: inception cohort study BMJ 2008 337 154 157 10.1136/bmj.a171 PMC2483884 18614473

[B10] Thomas E Silman AJ Croft PR Predicting who develops chronic low back pain in primary care BMJ 1999 318 1662 1667 10.1136/bmj.318.7199.1662 10373170 PMC28145

[B11] Carey TS Garrett JM Jackman AM Beyond good prognosis – examination of an inception cohort of patients with chronic low back pain Spine 2000 25 115 120 10.1097/00007632-200001010-00019 10647169

[B12] Jones GT Johnson RE Wiles NJ Chaddock C Potter RG Roberts C Predicting persistent disabling low back pain in general practice: a prospective cohort study Br J Gen Pract 2006 56 334 341 16638248 PMC1837841

[B13] Costa Lda C Maher CG McAuley JH Hancock MJ Herbert RD Refshauge KM Prognosis for patients with chronic low back pain: inception cohort study BMJ 2009 339 b3829 10.1136/bmj.b3829 19808766 PMC2758336

[B14] Hay EM Dunn KM Prognosis of low back pain in primary care BMJ 2009 339 816 817 10.1136/bmj.b3694 19808764

[B15] Sprouse R Treatment: current treatment recommendations for acute and chronic undifferentiated low back pain Prim Care 2012 39 481 486 10.1016/j.pop.2012.06.004 22958557

[B16] Giovannoni A Minozzi S Negrini S Percorsi diagnostico terapeutici per l’assistenza ai pazienti con mal di schiena 2006 Pacini Editore: Pisa

[B17] Deutsche Gesellschaft für Allgemeinmedizin und Familienmedizin (DEGAM) Leitlinie Kreuzschmerzen (Guideline: Low Back Pain) 2003 Duesseldorf: Omikron publishing

[B18] Piccoliori G Gatterer D Sessa E Abholz HH Der Kreuzschmerz in der Hausarztpraxis – Epidemiologie und Versorgung in Hausarztpraxen in Bozen. (Low back pain in general practice – epidemiology and care in general practice in Bozen/Italy) Z Allg Med 2007 83 285 291 10.1055/s-2007-984358

[B19] Becker A Breyer R Kölling W Sönnichsen A Donner-Banzhoff N Kreuzschmerzen in der Praxis: was tun Allgemeinärzte und was Orthopäden? (Low back pain in practice: what do general practitioners do and what orthopedists?) Z Allg Med 2007 83 44 50 10.1055/s-2007-970076

[B20] Sieben JM Vlaeyen JW Postegijs PJ Warmenhoven FC Sint AG Dautzenberg N General practitioners orientation toward low back pain: Influence on treatment behaviour and patient outcome Europ J Pain 2009 13 412 418 10.1016/j.ejpain.2008.05.002 18562224

[B21] Schers H Braspenning J Drijver R Wensing M Grol R Low back pain in general practice: reported management and reasons for not adhering to the guidelines in The Netherlands Br J Gen Pract 2000 50 640 644 11042916 PMC1313775

[B22] Fullen BM Maher T Bury G Tynan A Daly LE Hurley DA Adherance of Irish general practitioners to European guidelines for acute low back pain: a prospective pilot study Europ J Pain 2007 11 614 623 10.1016/j.ejpain.2006.09.007 17126046

[B23] Williams CM Maher CG Hancock MJ McAuley JH McLachlan AJ Britt H Low back pain and best practice care Arch Intern Med 2010 170 291 77 10.1001/archinternmed.2009.507 20142573

[B24] Ihlebaek C Eriksen HR The myths of low back pain: status quo in Norwegian general practitioners and physiotherapists Spine 2004 29 1818 1822 10.1097/01.BRS.0000134566.50519.65 15303028

[B25] Snyder L Neubauer RL Pay-for-performance principles that promote patient-centered care: an ethics manifesto Ann Intern Med 2007 147 792 794 10.7326/0003-4819-147-11-200712040-00011 18056664

[B26] Becker A Kögel K Donner-Banzhoff N Basler HD Chenot JF Maitra R Kreuzschmerzpatienten in der hausärztlichen Praxis: Beschwerden, Behandlungserwartung und Versorgungsdaten. (Low back pain – Complaints, expectations for treatment, and data on care) Z Allg Med 2003 79 126 131 10.1055/s-2003-39278

[B27] Konstantinou K Dunn KM Sciatica: a review of epidemiological studies and prevalence estimates Spine 2008 33 2464 2472 10.1097/BRS.0b013e318183a4a2 18923325

[B28] Bewig A Abholz HH Pille oder Spritze? Untersuchung zur Frage eines Unterschieds am Beispiel des akuten Rückenschmerzes. (Pill or injection? Randomized trial on differences in success in case of low back pain) Z Allg Med 2001 77 31 35

[B29] Schers H Wensing M Huijsmans Z van Tulder M Grol R Implementation barriers for general practice guidelines on low back pain: a qualitative study Spine 2001 26 E348 E353 10.1097/00007632-200108010-00013 11474367

[B30] Piccoliori G Abholz H-H Engl A Sessa E Mahlknecht JF Die Behandlung von Patienten mit chronischer Herzinsuffizienz in der Hausarztpraxis -Eine Erhebung an 693 konsekutiven Patienten in Südtiroler Hausarztpraxen. (Treatment of patients with chronic heart failure in Family medicine- A study with 693 consecutively enrolled patients in South-Tyrol, Northerrn Italy) Zeitschr Allg Med 2012 88 100 107

[B31] Aline Ramond A Boutona C Richardb J Roquelaure Y Baufretone C Legrand E Hueza J-F Psychosocial risk factors for chronic low back pain in primary care—a systematic review Fam Pract 2011 28 12 21 10.1093/fampra/cmq072 20833704

[B32] Altiner A Brockmann S Sielk S Wilm S Wegscheider K Abholz H-H Reducing antibiotic prescriptions for acute cough motivating family practitioners to change their attitudes to communication and empowering patients: a cluster-randomised intervention study J Antimicrob Chemother 2007 60 638 644 10.1093/jac/dkm254 17626023

